# Gallstone Disease Is Associated with More Severe Liver Damage in Patients with Non-Alcoholic Fatty Liver Disease

**DOI:** 10.1371/journal.pone.0041183

**Published:** 2012-07-25

**Authors:** Anna Ludovica Fracanzani, Luca Valenti, Maurizio Russello, Luca Miele, Cristina Bertelli, Alessandro Bellia, Chiara Masetti, Consuelo Cefalo, Antonio Grieco, Giulio Marchesini, Silvia Fargion

**Affiliations:** 1 Department of Internal Medicine, Centro Studi Malattie Metaboliche del Fegato, University of Milano, Maggiore Policlinico Hospital, IRCCS Ca’ Granda Foundation, Milano, Italy; 2 Liver Unit, ARNAS, Garibaldi Hospital, Catania, Italy; 3 Department of Internal Medicine, Cattolica University, Roma, Italy; 4 Department of Internal Medicine, Alma Mater Studiorum University, Bologna, Italy; University of Tor Vergata, Italy

## Abstract

**Background:**

Nonalcoholic fatty liver disease (NAFLD) and gallstone disease (GD) are both highly prevalent in the general population and associated with obesity and insulin resistance. We aimed to evaluate the prevalence of GD in a cross sectional study of NAFLD patients and to define whether the presence of GD is associated with diabetes and predicts more severe liver disease.

**Methodology/Principal Findings:**

We merged databases of four Liver Units, comprising 524 consecutive biopsy-proven NAFLD (373 males) observed between January 2003 and June 2010. GD was diagnosed in 108 (20%), and 313 cases (60%) were classified by liver biopsy as nonalcoholic steatohepatitis (NASH). The GD subgroup was characterized by a significantly higher prevalence of females, prediabetes/diabetes, abdominal obesity and metabolic syndrome, older age, higher BMI, fasting glucose, HOMA-IR and lower ALT. The prevalence of GD progressively increased with advancing fibrosis and with the severity of necroinflammatory activity (p for trend  = 0.0001 and  = 0.01, respectively), without differences in the severity of steatosis. At multivariate analysis GD was associated with female gender (OR 1.37, 95% CI 1.04–1.8), age (OR 1.027, 95% CI1.003–1.05), fasting glucose (OR 1.21, 95% CI 1.10–1.33) and NASH (OR 1.40,95% CI 1.06–1.89), whereas ALT levels were associated with a lower GD risk (OR 0.98, 95% CI 0.97–0.99). When subjects with cirrhosis were excluded from analysis, the association between GD and fasting glucose, female gender, and NASH was maintained.

**Conclusion:**

Patients with NAFLD have a high prevalence of GD, which characterizes subjects with altered glucose regulation and more advanced liver disease.

## Introduction

Nonalcoholic fatty liver disease (NAFLD) includes a wide spectrum of liver conditions ranging from pure fatty liver, usually a benign and non-progressive condition, to non-alcoholic steatohepatitis (NASH), which may eventually progress to cirrhosis, portal hypertension and hepatocellular carcinoma [1], [2], [3]. Hepatic insulin resistance, associated with obesity, type 2 diabetes and dyslipidemia, is the underlying metabolic milieu favoring the occurrence of NAFLD [4], also accounting for the risk of progressive liver disease observed in these patients. NAFLD is now considered the hepatic expression of the metabolic syndrome [3], [4], [5], [6]; as such it is also associated with a high risk of cardiovascular disease [7], [8], [9].

Gallstone disease (GD) is one of the most common disorders of the gastrointestinal tract. Overall, the prevalence of gallstones in the general population is approximately 10–15% in the U.S. [10] and 9.5–18.9 in Italy [11]. The very high prevalence of both GD and NAFLD makes it very likely a chance co-occurrence in a high number of cases, but NAFLD and GD also share common risk factors. Both diseases are associated with overweight/obesity, hypertriglyceridemia, insulin resistance, and type 2 diabetes mellitus, and their coexistence might also be pathogenically mediated [12], [13], [14].

Recent experimental studies report that hepatic insulin resistance may be associated with biliary cholesterol secretion, thus promoting cholesterol gallstone formation [15], [16]. Hyperinsulinemia may increase hepatic cholesterol secretion, biliary cholesterol super-saturation and gallbladder dysmotility, all favoring gallstone formation, whereas insulin resistance has been associated with GD even in non-diabetic, non-obese individuals [17], thus providing a link between the metabolic syndrome and gallstone susceptibility.

## Methods

### Objectives

Aim of the present study was to evaluate the prevalence of GD and associated factors in a large series of patients with histologically proven NAFLD and to define whether the presence of GD is associated with type 2 diabetes and predicts more severe liver disease of metabolic origin.

### Patients

We merged the databases of four Liver Units, all referral centers for metabolic liver disease, long working together with shared protocols [18], [19]. The Case Report Form used by the centers was the basis of a collaborative Italian study sponsored by the Italian Association for the Study of the Liver on Liver Fat (Steatosi Epatica - STEP study). Consecutive patients submitted to a diagnostic liver biopsy between January 2003 and June 2010 were included in the study, provided that the tissue sample size was ≥1.7 cm. No cases had liver biopsy during cholecistectomy.

The final cohort comprised 524 NAFLD cases, in which all other causes of liver diseases (viral, autoimmune, drug-induced, hereditary hemochromatosis, Wilson’s disease) had been excluded and whose daily alcohol intake, confirmed by at least one family member, was ≤20 g. Liver biopsy had been performed in the presence of long-lasting elevation of liver enzymes and/or ferritin, coupled with a long history of steatosis [18]. Cases diagnosed as fibrosis stage 4 at histology [20], [21] were considered as having cirrhosis, but were maintained in the analysis. By contrast, subjects with clinical or sonographic evidence of decompensated cirrhosis and a limited number (5 cases) of subjects of non-Caucasian origin were excluded.

### Ethics

The observational study protocol was first approved by the Institutional Review Board (IRB) of ‘‘Ca’ Granda’’ IRCCS Foundation, Ospedale Maggiore Policlinico, Milan, Italy, regulating the activity of the Liver unit that designed the study and coordinated data collection, while for the other Liver Units, once approval of the proponent center was obtained only a notification of the study to the IRBs local Institutions was required. Written consent was provided by patients for data storing in the hospital database and use for research. The study conforms to the ethical guidelines of the 1975 declaration of Helsinki.

### Methods

At enrollment lifestyle habits, clinical and pharmacological history, BMI, waist circumference (measured at the level of the umbilicus, at the midlevel between the lowest rib and the iliac crest, with the subject standing and breathing normally) and arterial blood pressure (defined as the mean of the second and third reading of three consecutive blood pressure measurements), complete blood count, alanine and aspartate aminotransferase (ALT-AST) gamma-glutamyltransferase (GGT), serum alkaline phosphatase (SAP) and bilirubin, fasting glucose, total and HDL cholesterol, triglycerides, and uric acid, were available in all subjects, determined by determined standard laboratory procedures and insulin had been measured by a commercially purchased radioimmunoassay (RIA, Biochem Immunosystems, Bologna, Italy), used in the participating Units. The metabolic syndrome was diagnosed according to the presence of 3 or more of the revised ATPIII criteria [22].

Insulin resistance was evaluated according to the homeostatic model assessment (HOMA) [23]. An oral 75 g glucose tolerance test (OGTT) had been performed in all subjects without diabetes according to World Health Organization criteria. The presence of diabetes (fasting glucose ≥7 mmol/l, 120-min glucose during OGTT ≥11.1 mmol/l, or treatment with glucose-lowering drugs), obesity (BMI >30 kg/m^2^) and overweight (BMI, 25–29.9 kg/m^2^) were also recorded.

### Liver Histology

Liver biopsies were processed routinely, and read by a single pathologist in each centre. To control for biopsy size, the length of the biopsy was measured with a hand ruler, and the number of portal areas on a cross-section was counted. The minimum biopsy size for inclusion in the study was 1.7 cm and the number of portal areas was 10. Steatosis was graded 1 to 3 according to the percentage of cells with fatty droplets (grade 1: 10–33%, 2: 33–66% and 3>66%). NASH was diagnosed by experienced pathologists according to standard criteria; their agreement was checked in this as in other studies as part of common granted protocols.

### Definition of Gallstone Disease (GD)

Ultrasonography had been performed *per protocol* for the evaluation of steatosis at time of biochemistry evaluation. Gallstone disease was diagnosed in the presence of one of the following criteria: (i) sonographic evidence of gallstones (one or more echogenic, distally shadowing, possibly movable structures in the gallbladder); (ii) echogenic material within the gallbladder with constant shadowing and little or no visualization of the gallbladder; and (iii) absence of gallbladder at ultrasonography, coupled with a history of cholecystectomy (in 14 cases).

### Statistical Analysis

Results are expressed as means ± standard deviations for continuous variables and as frequencies for categorical variables. Mean values were compared by t-test for unequal variances. Frequencies were compared by chi-square test. P values ≤0.05 were considered statistically significant. Continuous variables were correlated by Spearman test. Logistic regression analyses were performed to assess the variables independently associated with the presence of NASH and moderate-to-severe fibrosis (stage ≥2). This cut-off was chosen because of the relatively mild fibrosis detected in our patients. Variables significant at univariate analyses were entered in a multivariate analysis. All statistical analyses were performed by the JMP statistical discovery software system (SAS, Cary, NC).

## Results

GD was diagnosed in 107/524 (20%) NAFLD patients by sonographic scanning and in 14 (13%) on the basis of previous cholecystectomy.

**Figure 1:**
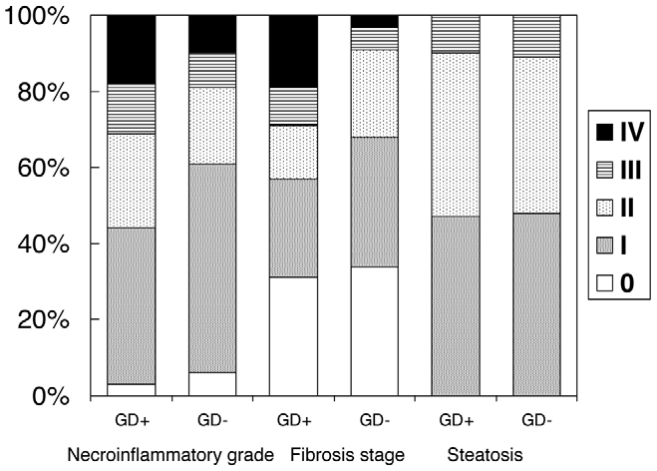
Percentage of necro inflammatory grading (left), fibrosis staging (middle) and steatosis (right) according to the presence or absence of GD. For necroinflammatory grade (IV  =  grade IV, V, VI and VII together). Analysis for trend: Necroinflammatory grade p = 0.01, Fibrosis stage p = 0.0001, Steatosis not significant (p = 0.7).

At histology, 343 cases (65.5%) had minimum (stage 1) or no fibrosis, 111 had fibrosis stage 2 (21.5%), 37 had fibrosis stage 3 (7.1%) and 33 (6.3%) were classified as cirrhosis (stage 4 fibrosis). The prevalence of GD progressively increased with advancing fibrosis from about 15% (stage 0–2) to 29% (stage 3) and 56% (stage 4), and with the severity of necro-inflammatory activity from 14% (grade 0–1), to 23% (grade 2–3), 28% (grade 4) and 35% (grade >4), (p for trend  = 0.0001 and  = 0.01, respectively), without differences in the severity of steatosis (Figure 1).

Three hundred and sixteen cases were diagnosed with NASH, and the prevalence of NASH was significantly higher in the presence of GD (82/107 (77%) vs. 231/417 (56%) without GD, p = 0.0001).

One hundred forty-four patients had diabetes. In 106 cases, the diagnosis preceded NAFLD (by 77±70 months), in the remaining 38 the diagnosis was contemporary to that of NAFLD. Mean HbA1c was 7.2±4.8% without difference between patients with or without GD. In patients with a known history of diabetes HbA1c was higher. Of the 106 patients with a previous diagnosis of diabetes, 27 were treated with insulin, 47 with oral hypoglycaemic agents and 32 with diet only. Twenty-eight cases with diabetes had cirrhosis.

Compared with cases with no GD, the GD group was characterized by a significantly higher prevalence of female gender, prediabetes/diabetes, abdominal obesity, metabolic syndrome, older age, as well as higher BMI, fasting glucose, HOMA-IR, higher AST/ALT ratio and lower ALT (table 1). After exclusion of subjects with cirrhosis, the presence of GD remained significantly associated with female gender (OR 1.45; 95% CI 1.13–1.85), age (OR 14, 95% CI 4.1–51.2), AST/ALT (OR 2.6, 95% CI 1.33–5.2), ALT (OR 0.99, 95% CI 0.98–0.99), fasting glucose (OR 1.19, 95% CI 1.06–1.32), presence of MS (OR 1.29, 95% CI 1.07–1.65) and NASH (OR 1.47 95% CI 1.15–1.92). In particular, the presence of prediabetes (impaired fasting glucose and/or the presence of impaired glucose tolerance) and diabetes remained significantly associated with GD (OR 1.27, 95% CI 1.06–1.61; OR 1.27, 95% CI 1.006–1.61, respectively).

**Table 1:** Anthropometric, biochemical and clinical variables in patients with NAFLD with or without GD (univariate analysis).

Variables	GD (n = 107)	non GD (n = 417)	P value+	OR for GD	95% CI
Female gender*	48 (45%)	103 (25%)	0.0001	1.57	1.26–1.96
Age* (yrs)	54±12	47±13	0.0001	1.05	1.03–1.07
BMI (kg/m^2^)	29.6±5.2	28.1±4.6	0.01	1.06	1.01–1.11
Overweight/obesity (BMI ≥25 kg/m^2^)	91 (85%)	316 (76%)	0.05	1.7	1.01–3.3
Large waist circumference (male >102, female >88 cm)	68 (64%)	15 (36%)	0.02	1.46	1.12–1.94
Fasting glucose* (mmol/L)	6.8±3.0	5.8±1.7	0.001	1.21	1.1–1.301
Prediabetes (fasting glucose ≥5.5 mmol/L and or impaired glucose tolerance)*	59 (55%)	150 (36%)	0.0003	1.49	1.20–1.86
Diabetes*	44 (41%)	11 (26%)	0.002	1.82	1.13–78
HOMA-IR	6.2±6.1	4.6±4.3	0.03	1.05	1.003–1.11
Triglycerides (mg/dL)	146±77	146±81	0.1	0.99	0.99–1.00
Triglycerides ≥150 (mg/dL)	43 (40%)	154 (37%)	0.5	0.93	0.94–1.17
Total cholesterol (mg/dL)	196±42	201±44	0.2	0.99	0.99–1.00
HDL-cholesterol (mg/dl)	48±14	45±12	0.99	0.99	0.99–1.00
Males	47±13	44±12	0.1	1.00	0.99–1.0
Females	50±16	48±12	0.6	1.01	0.97–1.04
Serum ferritin (ng/ml)	250±208	288±277	0.1	0.99	0.99–1.01
ALT* (U/L)	51±28	65±40	0.0001	0.88	0.91–0.94
AST/ALT*	0.84±0.4	0.71±0.31	0.0001	2.0	1.0–4.0
Arterial hypertension	43 (40%)	158(38%)	0.8	0.98	0.77–1.02
Metabolic syndrome*	54 (50%)	133 (31%)	0.0007	1.48	1.17–1.85
NASH*	83 (77%)	233 (56%)	0.0001	1.64	1.29–2.11

Mean ± SD or number of cases and (%).

+ P-values (ANOVA or chi-square test).

* Variables that maintained significance when the subjects diagnosed with cirrhosis were excluded from analysis.

In the overall series lipid parameters (total and HDL cholesterol, triglycerides, analyzed both as continuous and as categorical variables), bilirubin and other liver enzymes did not significantly differ according to the presence of GD. No difference was also observed between patients with GD diagnosed at the time of NAFLD assessment and those with a diagnosis of GD based on previous cholecystectomy.

At multivariate analysis (table 2), the variables independently associated with GD were female gender, age, fasting glucose and NASH, whereas ALT levels were associated with a lower risk. When the 33 subjects with cirrhosis were excluded from the analysis, GD was significantly associated with fasting glucose, female gender and NASH. Excluding from the analysis the 144 patients with diabetes, NASH remained the only independent variable associated with GD.

**Table 2:** Adjusted risks associated with GD in the overall series, in patients without cirrhosis and without diabetes, at multivariate logistic regression analysis.

	Patients All		Patients without cirrhosis		Patients without diabetes	
Variables	OR adj	95% CI	OR adj	95% CI	OR adj	95% CI
Female gender	1.44	1.07–1.92	1.44	1.06–1.47	1.30	0.96–1.85
Age (yrs)	1.03	1.00–1.04	1.01	1.00–1.04	1.05	1.01–1.07
Overweight/obesity (BMI >25 kg/m^2^)	1.05	0.73–1.48	0.95	0.67–1.30	1.01	0.84–1.09
Waist circumference (M >102, F >88 cm)	1.00	0.96–1.34	1.00	0.97–1.03	0.98	0.93–1.03
Fasting glucose (mmol/L)	1.15	1.00–1.35	1.18	1.00–1.10	1.06	0.98–1.03
Diabetes	1.24	0.83–1.86	0.75	0.48–1.13	–	–
HOMA-IR	1.18	0.82–1.74	1.03	0.96–1.10	1.02	0.83–1.87
ALT U/L	0.98	0.97–0.99	0.98	0.97–0.99	0.99	0.98–1.00
AST/ALT	1.15	0.80–2.70	1.69	0.73–3.84	0.33	0.6–1.07
Metabolic Syndrome	1.16	0.84–1.59	1.09	0.81–1.49	1.2	0.89–1.78
NASH	1.46	1.08–1.99	1.51	1.14–2.15	1.5	1.10–2.10

Each variable adjusted for the others in the table.

## Discussion

In this large series of well-characterized Italian patients with biopsy-proven NAFLD we provide evidence for an association of GD with glucose alterations, NASH and severe fibrosis, without difference in the severity of steatosis.

A few papers have already suggested the existence of an association between GD and NAFLD [24], [25]. This association might stem from the pathogenic factors shared by both GD and NAFLD, given that the risk for GD is especially high in patients with central obesity, type 2 diabetes and insulin resistance [12], [13], [14], [26], [27]. All these conditions are components of the metabolic syndrome and NAFLD itself is considered the hepatic expression of the metabolic syndrome. However, despite the association of NASH and GD was tentatively related to central obesity [28], BMI and large waist circumference were not significantly associated with GD at multivariate analysis.

The strength of the study is the very large sample size of well-characterized, biopsy proven NAFLD patients, which provided a reasonable number of GD cases to characterize risk factors for GD in relation to histology. The overall prevalence of GD in our series was 20%, a value similar to that previously reported in another Italian NAFLD series [24] and slightly higher than in the general population (3.3–14.8%) [10], [11]. As expected, GD remained significantly associated with female gender. The prevalence of GD was higher in subjects with the metabolic syndrome, in keeping with the results reported in the general population and in obese subjects [29]. The metabolic syndrome was significantly associated with GD at univariate analysis, even when adjusted for gender and age, but the significance was lost at multivariate analysis, at variance from data reported by Mendez-Sanchez et al [30] in a smaller series.

Of note, fasting glucose level, not the presence of diabetes, remained significantly associated with GD at multivariate analysis. In a population-based case-control analysis of the UK General Practice Research Database (22,574 cases with cholecystectomy and 72,476 controls), Bodmer et al concluded that neither the presence or duration of diabetes, nor metabolic control or use of oral antidiabetic therapies were associated with an altered risk of cholecystectomy [31]. In contrast, the risk of biliary disease (defined as occurrence of cholelithiasis, acute cholecystitis, or cholecystectomy) was reported to be 1.9-fold increased in a large retrospective analysis of several U.S. health care claims databases of patients with type 2 diabetes [32]. Differences might also derive from different rates of statin use in NAFLD (8% of cases in our series, with no difference in relation to the presence/absence of GD), considering that a protective effect of statins on GD has been reported in patients with and without diabetes [31], an effect that might also extend to protection against NASH [33] .

From a pathogenic perspective, hepatic insulin resistance, the metabolic milieu of steatosis, is associated with biliary cholesterol secretion, a mechanism to explain cholesterol gallstone formation [34]. Clinical studies confirmed that insulin resistance could have a major role in the pathogenesis of GD, by favoring the production of cholesterol-supersaturated bile and by altering gallbladder function [17]. However in our series we found an association of GD with HOMA only at univariate, not at multivariate analysis, possibly because of the high proportion of patients with diabetes and the wide range of glucose levels, entering the predictive model and limiting the significance of HOMA-IR. No association with serum bilirubin and alkaline phosphatase levels was detected, indirectly excluding the presence of bile duct stones as a contributing factor to more severe liver disease in patients with GD.

Unexpectedly, ALT levels were significantly lower in patients with GD, either when ALT were analyzed as continuous variables or when categorized as values above normal range, although GD was significantly associated with more severe histological liver damage. A poor prognostic significance of elevated ALT has been reported in several series of NAFLD patients [18], [35], but it remains difficult to explain these finding on a mechanicistic base. GD is not systematically associated with raised ALT [36], [37]. Interestingly Ioannou et al [36], analyzing cohort data from the first National Health and Nutrition Examination Survey (NHANES), found that cholelithiasis was independently associated with cirrhosis, not with serum liver enzymes. In our series, the higher necroinflammatory activity observed in GD would be expected to be associated with a risk of raised liver enzymes, but the pattern of aminotransferases levels changes with progressive disease and steatosis tend to disappear in more advanced NAFLD. This observation requires additional studies.

Our study has clinical relevance. About half of the patients with GD undergoing liver biopsy at the time of cholecystectomy were reported to have NAFLD [25], suggesting the opportunity of a routine liver biopsy during surgery to establish an early diagnosis of NAFLD, a policy supported by a NHANES report [36] and by a recent EASL Consensus for liver biopsy in NAFLD [38].

### Limitations and Strengths

Major limitations of the study are the small group of cases with previous cholecistectomy, which did not allow evaluate whether these patients have different hepatic outcome. Similarly, the low number of patients with cirrhosis did not permit a separate analysis of risk factors in this cohort. The strength of the study is the large number of patients with biopsy proven NAFLD who were evaluated for GD.

In conclusion, although the cross-sectional nature of the study does not allow to completely understand the cause-effect relationship between GD and NASH, the presence of GD in a subject with steatosis deserves consideration as a possible risk of liver disease severity. A liver biopsy in these cases should always be considered, because of the higher risk of NASH and severe fibrosis. Cases with hyperglycemia/diabetes might be worth a more careful scrutiny.

## References

[1] Angulo P (2002). Nonalcoholic fatty liver disease.. N Engl J Med.

[2] Bugianesi E, Leone N, Vanni E, Marchesini G, Brunello F (2002). Expanding the natural history of nonalcoholic steatohepatitis: From cryptogenic cirrhosis to hepatocellular carcinoma.. Gastroenterology.

[3] Vernon G, Baranova A, Younossi ZM (2011). Systematic review: the epidemiology and natural history of non-alcoholic fatty liver disease and non-alcoholic steatohepatitis in adults.. Aliment Pharmacol Ther.

[4] Adams LA, Waters OR, Knuiman MW, Elliott RR, Olynyk JK (2009). NAFLD as a risk factor for the development of diabetes and the metabolic syndrome: an eleven-year follow-up study.. Am J Gastroenterol.

[5] Marchesini G, Brizi M, Bianchi G, Tomassetti S, Bugianesi E (2001). Nonalcoholic fatty liver disease: a feature of the metabolic syndrome.. Diabetes.

[6] Younossi ZM (2008). Review article: current management of non-alcoholic fatty liver disease and non-alcoholic steatohepatitis.. Aliment Pharmacol Ther.

[7] Fracanzani AL, Burdick L, Raselli S, Pedotti P, Grigore L (2008). Carotid artery intima-media thickness in nonalcoholic fatty liver disease.. Am J Med.

[8] Targher G, Day CP, Bonora E (2010). Risk of cardiovascular disease in patients with nonalcoholic fatty liver disease.. N Engl J Med.

[9] Yilmaz Y, Kurt R, Yonal O, Polat N, Celikel CA (2010). Coronary flow reserve is impaired in patients with nonalcoholic fatty liver disease: association with liver fibrosis.. Atherosclerosis.

[10] Everhart JE, Khare M, Hill M, Maurer KR (1999). Prevalence and ethnic differences in gallbladder disease in the United States.. Gastroenterology.

[11] Attili AF, Carulli N, Roda E, Barbara B, Capocaccia L (1995). Epidemiology of gallstone disease in Italy: prevalence data of the Multicenter Italian Study on Cholelithiasis (M.I.COL.).. Am J Epidemiol.

[12] De Santis A, Attili AF, Ginanni Corradini S, Scafato E, Cantagalli A (1997). Gallstones and diabetes: a case-control study in a free-living population sample.. Hepatology.

[13] Diehl AK (2000). Cholelithiasis and the insulin resistance syndrome.. Hepatology.

[14] Ruhl CE, Everhart JE (2000). Association of diabetes, serum insulin, and C-peptide with gallbladder disease.. Hepatology.

[15] Tsai CJ, Leitzmann MF, Willett WC, Giovannucci EL (2008). Macronutrients and insulin resistance in cholesterol gallstone disease.. Am J Gastroenterol.

[16] Xie Y, Newberry EP, Kennedy SM, Luo J, Davidson NO (2009). Increased susceptibility to diet-induced gallstones in liver fatty acid binding protein knockout mice.. J Lipid Res.

[17] Chang Y, Sung E, Ryu S, Park YW, Jang YM (2008). Insulin resistance is associated with gallstones even in non-obese, non-diabetic Korean men.. J Korean Med Sci.

[18] Fracanzani AL, Valenti L, Bugianesi E, Andreoletti M, Colli A (2008). Risk of severe liver disease in NAFLD with normal aminotransferase levels: A role for insulin resistance and diabetes.. Hepatology.

[19] Fracanzani AL, Valenti L, Bugianesi E, Vanni E, Grieco A (2011). Risk of nonalcoholic steatohepatitis and fibrosis in patients with nonalcoholic fatty liver disease and low visceral adiposity.. J Hepatol.

[20] Kleiner DE, Brunt EM, Van Natta M, Behling C, Contos MJ (2005). Design and validation of a histological scoring system for nonalcoholic fatty liver disease.. Hepatology.

[21] Brunt EM (2001). Nonalcoholic steatohepatitis: definition and pathology.. Semin Liver Dis.

[22] Grundy SM, Brewer HB, Cleeman JI, Smith SC, Lenfant C (2004). Definition of metabolic syndrome: Report of the National Heart, Lung, and Blood Institute/American Heart Association conference on scientific issues related to definition.. Circulation.

[23] Matthews DR, Hosker JP, Rudenski AS, Naylor BA, Treacher DF (1985). Homeostasis model assessment: insulin resistance and beta-cell function from plasma fasting glucose and insulin concentrations in man.. Diabetologia.

[24] Loria P, Lonardo A, Lombardini S, Carulli L, Verrone A (2005). Gallstone disease in non-alcoholic fatty liver: prevalence and associated factors.. J Gastroenterol Hepatol.

[25] Ramos-De la Medina A, Remes-Troche JM, Roesch-Dietlen FB, Perez-Morales AG, Martinez S (2008). Routine liver biopsy to screen for nonalcoholic fatty liver disease (NAFLD) during cholecystectomy for gallstone disease: is it justified?. J Gastrointest Surg 12: 2097–2102; discussion 2102.

[26] Olokoba AB, Bojuwoye BJ, Olokoba LB, Braimoh KT, Inikori AK (2007). Gallstone disease and type-2 diabetes mellitus-the link.. J Coll Physicians Surg Pak.

[27] Tsai CJ, Leitzmann MF, Willett WC, Giovannucci EL (2006). Central adiposity, regional fat distribution, and the risk of cholecystectomy in women.. Gut.

[28] Liew PL, Lee WJ, Wang W, Lee YC, Chen WY (2008). Fatty liver disease: predictors of nonalcoholic steatohepatitis and gallbladder disease in morbid obesity.. Obes Surg.

[29] Shaffer EA (2006). Gallstone disease: Epidemiology of gallbladder stone disease.. Best Pract Res Clin Gastroenterol.

[30] Mendez-Sanchez N, Chavez-Tapia NC, Motola-Kuba D, Sanchez-Lara K, Ponciano-Rodriguez G (2005). Metabolic syndrome as a risk factor for gallstone disease.. World J Gastroenterol.

[31] Bodmer M, Brauchli YB, Jick SS, Meier CR (2011). Diabetes mellitus and the risk of cholecystectomy.. Dig Liver Dis.

[32] Noel RA, Braun DK, Patterson RE, Bloomgren GL (2009). Increased risk of acute pancreatitis and biliary disease observed in patients with type 2 diabetes: a retrospective cohort study.. Diabetes Care.

[33] Tsai CJ, Leitzmann MF, Willett WC, Giovannucci EL (2009). Statin use and the risk of cholecystectomy in women.. Gastroenterology.

[34] Biddinger SB, Haas JT, Yu BB, Bezy O, Jing E (2008). Hepatic insulin resistance directly promotes formation of cholesterol gallstones.. Nat Med.

[35] Bhala N, Angulo P, van der Poorten D, Lee E, Hui JM (2011). The natural history of nonalcoholic fatty liver disease with advanced fibrosis or cirrhosis: an international collaborative study.. Hepatology.

[36] Ioannou GN (2010). Cholelithiasis, cholecystectomy, and liver disease.. Am J Gastroenterol.

[37] Padda MS, Singh S, Tang SJ, Rockey DC (2009). Liver test patterns in patients with acute calculous cholecystitis and/or choledocholithiasis.. Aliment Pharmacol Ther.

[38] Ratziu V, Bellentani S, Cortez-Pinto H, Day C, Marchesini G (2010). A position statement on NAFLD/NASH based on the EASL 2009 special conference.. J Hepatol.

